# *DNAH11* and a Novel Genetic Variant Associated with Situs Inversus: A Case Report and Review of the Literature

**DOI:** 10.1155/2023/8436715

**Published:** 2023-04-25

**Authors:** Fatemeh Sodeifian, Noosha Samieefar, Sepideh Shahkarami, Elham Rayzan, Simin Seyedpour, Meino Rohlfs, Christoph Klein, Delara Babaie, Nima Rezaei

**Affiliations:** ^1^Student Research Committee, School of Medicine, Shahid Beheshti University of Medical Sciences, Tehran, Iran; ^2^Network of Interdisciplinarity in Neonates and Infants (NINI), Universal Scientific Education and Research Network (USERN), Tehran, Iran; ^3^USERN Office, Shahid Beheshti University of Medical Sciences, Tehran, Iran; ^4^Department of Pediatrics, Dr. von Hauner Children's Hospital, University Hospital, Ludwig-Maximilians-Universität München (LMU), Munich, Germany; ^5^International Hematology/Oncology of Pediatric Experts (IHOPE), Universal Scientific Education and Research Network (USERN), Boston, Massachusetts, USA; ^6^MD-MPH, School of Medicine, Tehran University of Medical Sciences, Tehran, Iran; ^7^Research Center for Immunodeficiencies (RCID), Tehran, Iran; ^8^Department of Allergy and Clinical Immunology, Mofid Children Hospital, Shahid Beheshti University of Medical Sciences, Tehran, Iran; ^9^Research Center for Immunodeficiencies, Pediatrics Center of Excellence, Children's Medical Center, Tehran University of Medical Sciences, Tehran, Iran

## Abstract

**Background:**

Primary ciliary dyskinesia (PCD), also known as the immotile-cilia syndrome, is a clinically and genetically heterogeneous syndrome. Improper function of the cilia causes impaired mucociliary clearance. Neonatal respiratory distress, rhinosinusitis, recurrent chest infections, wet cough, and otitis media are respiratory presentations of this disease. It could also manifest as infertility in males as well as laterality defects in both sexes, such as situs abnormalities (Kartagener syndrome). During the past decade, numerous pathogenic variants in 40 genes have been identified as the causatives of primary ciliary dyskinesia. *DNAH11* (dynein axonemal heavy chain 11) is a gene that is responsible for the production of cilia's protein and encodes the outer dynein arm. Dynein heavy chains are motor proteins of the outer dynein arms and play an essential role in ciliary motility. *Case Presentation*. A 3-year-old boy, the offspring of consanguineous parents, was referred to the pediatric clinical immunology outpatient department with a history of recurrent respiratory tract infections and periodic fever. Furthermore, on medical examination, situs inversus was recognized. His lab results revealed elevated levels of erythrocyte sedimentation rate (ESR) and C reactive protein (CRP). Serum IgG, IgM, and IgA levels were normal, while IgE levels were elevated. Whole exome sequencing (WES) was performed for the patient. WES demonstrated a novel homozygous nonsense variant in *DNAH11* (c.5247G > A; p. Trp1749Ter).

**Conclusion:**

We reported a novel homozygous nonsense variant in *DNAH11* in a 3-year-old boy with primary ciliary dyskinesia. Biallelic pathogenic variants in one of the many coding genes involved in the process of ciliogenesis lead to PCD.

## 1. Background

Axonemal structure dysfunction has been identified as being related to the emerging classes of disorders collectively known as “ciliopathies.” Examples include primary ciliary dyskinesia (PCD)/Kartagener syndrome, Bardet–Biedl syndrome, hydrocephalus, polycystic kidney disease, polycystic liver disease, nephronophthisis, Meckel–Gruber syndrome, Joubert syndrome, Alstrom syndrome, Jeune syndrome, laterality defects, etc. Primary ciliary dyskinesia, also known as the immotile-cilia syndrome, is a heterogeneous, mostly autosomal recessive genetic disorder and the first human disorder related to the motility dysfunction of cilia. Impaired mucociliary function due to defective axonemal structure leads to PCD recurrent respiratory manifestations (chronic bronchitis, chronic rhinosinusitis, otitis media, and pneumonia), male infertility, as well as laterality defects such as situs abnormalities [1]. Autosomal dominant variants of PCD have also been reported in *FOXJ1*-linked ciliary dyskinesia due to de-novo mutations (OMIM 618699, 602291) [2]. Over 300 genes have been identified to be involved in the function and morphology of the cilia, and at least 40 genes are involved in the inappropriate function of the cilia [3]. Dynein axonemal heavy chain 5 (*DNAH5*) and dynein axonemal intermediate chain 1 (*DNAI1*) are the most disease-causing genes involved in the pathogenesis of PCD [1]. *DNAH11* (dynein axonemal heavy chain 11) is a gene, which is responsible for the production of cilia's protein and encodes the outer dynein arm. Dynein heavy chains are motor proteins of the outer dynein arms and play an essential role in ciliary motility. Patients with pathogenic variants in this gene present symptoms associated with PCD but have normal cilia ultrastructure. The clinical manifestations of *DNAH11* pathogenic variants are usually similar to those of other variants causing PCD. The development of chronic middle ear infections and rhinosinusitis is the prominent feature of this disorder during childhood. In adulthood, the same symptoms persist; in addition, some radiological findings such as consolidations, atelectasis, and bronchiectasis might be discovered in high-resolution computed tomography (HRCT) of these patients. Moreover, bronchial colonization of *Pseudomonas aeruginosa* might be present [4].

Motile cilia can be found on the apical surfaces of the upper and lower respiratory tract, ependymal cells of CNS ventricles, oviducts (female reproductive system), and the flagellum of spermatozoa (male reproductive system) [5]. Respiratory cilia consist of a central pair of singlet microtubules surrounded by nine microtubule pairs, which make a cross-sectional 9 + 2 arrangement [6]. Proteins responsible for appropriate ciliary beating include inner and outer dynein arms that cause microtubules to slide through, generating ATP. The absence of the outer dynein arms, missed central pairs, and disarrangement of the microtubules are the ultrastructural defects causing PCD [7].

Over the past 15 years, our understanding of effective clinical care for PCD has progressed remarkably. Although respiratory manifestations of PCD present early in life, no effective treatment is available to stop the disease progression. Available treatments for PCD are extrapolated from other diseases, including cystic fibrosis (CF) and non-CF bronchiectasis [8].

In this study, we describe a patient who was investigated for PCD through genetic testing that eventually resulted in the reporting of a novel mutation in *DNAH11*.

## 2. Case Presentation

A 3-year-old boy, the offspring of consanguineous parents (first cousin), was referred to the pediatric immunology outpatient department with a history of recurrent respiratory tract infection, growth retardation, periodic fever, persistent rhinorrhea, and wheezing. Also, his history revealed allergies to different foods. In his referral letter, mild bronchiectasis in CT scan was also noted.

He had a history of hospital admission about three months before the visit, with the chief complaints of fever and rhinorrhea. Furthermore, on medical examination, situs inversus was recognized (the heart was on the right, the liver was on the left, and the spleen was located on the right side of the body). His lab results revealed elevated levels of erythrocyte sedimentation rate (ESR), C reactive protein (CRP), and white blood cell count (WBC) (see Table 1 for other lab data). The patient had normal levels of serum IgG, IgM, and IgA and elevated levels of serum IgE. A sweat test was done for possible cystic fibrosis (CF), and the sodium and chloride levels were 38 and 35, respectively (normal range: 5–35, borderline: 35–60, and for CF: 60–200). In addition, levels of antibodies against diphtheria and tetanus were in the normal range.

In the patient's follow-up, he had no other complications or complaints. However, the patient was overweight with an increased level of triglycerides and cholesterol. He is receiving metformin, diet therapy, and medications for allergies (montelukast and sprays).

## 3. Genetic Testing

Whole exome sequencing (WES) was performed for the patient at the Dr. von Hauner Children's Hospital NGS facility using Agilent V6 + UTR library preparation and an Illumina NextSeq 500 sequencing platform on *DNAH11* (Transcript: NM_001277115.2/Chromosome: 7p15.3-7p15.3). The bioinformatics analysis pipeline used Burrows-Wheeler Aligner (BWA 0.7.15), genome analysis toolkit (GATK 3.6), variant effect predictor (VEP 89), and frequency filters with public and in house databases (e.g. ExAC, GnomAD, and GME). WES demonstrated a novel homozygous nonsense variant in *DNAH11* (ENST00000409508: c.5247G > A) or p. Trp1749Ter (GRCh37.p13: 7:21698568 G->A stop).

## 4. Discussion and Conclusion

We reported a novel homozygous nonsense variant in *DNAH11* in a 3-year-old boy with primary ciliary dyskinesia born to a consanguineous family of Iranian descent. Our patient showed recurrent upper respiratory infections (URI), fever, and rhinorrhea, which were not responsive to typical medications prescribed for URI. Recurrent URI, born to a consanguineous parent, and situs inversus raised the susceptibility of genetic disorders such as primary immunodeficiencies. Due to the accessibility of genetic testing with an extended panel, we did not use any other tests to discover the pathology of the clinical manifestation. Furthermore, considering abnormal levels of serum immunoglobulins and blood cells, suspicion of genetic disorders was raised.

In the absence of prematurity and cystic fibrosis, a neonatal history of respiratory distress (pneumonia, cough, and tachycardia), rhinorrhea, otitis media, cough, and lateral defects should raise clinical suspicion of PCD, as it was manifested in our patient [9] (see Table 2 to check the clinical presentation of PCD).

PCD is a heterogeneous genetic disease that is caused by pathogenic variants in proteins essential for cilia assembly and function. In the majority of patients, the inheritance pattern of PCD transmission is autosomal recessive; however, *X*-linked inheritance patterns have also been revealed [11]. Several genes have been identified to cause PCD. Dynein axonemal intermediate chain 1 (*DNAI1*), *DNAI2*, dynein axonemal heavy chain 5 (*DNAH5*), *DNAH11*, and thioredoxin domain containing 3 (*TXNDC3*) encode components of the outer dynein Arm (ODA)[10]. Mutation of *DNAH11*, the first PCD gene identified in 1999, causes up to 14% of PCD cases. *DNAI1* encodes intermediate dynein chain 1 of ODA*. DNAH5,* the second identified gene associated with PCD, explains more than 25% of PCD cases and encodes the major molecular motor of ODA, called heavy dynein chain 5 [5]. Chromosome 14 open reading frame 104 gene (*KTU*) is responsible for cytoplasmic preassembly of axonemal dynein. Pathogenic variants in KTU have been reported to cause ciliary immotility and the absence of both dynein arms. Furthermore, pathogenic variants in the radial spoke head 9 homologue (*RSPH9*) and 4 homologues A (*RSPH4A*) genes lead to central microtubular pair abnormalities [10]. Pathogenic variants in the retinitis pigmentosa guanosine triphosphatase regulator gene (*RPGR*), which play an important role in the maintenance of photoreceptors, are associated with *X*-linked recessive retinitis pigmentosa, dysfunction of sensory hearing, and PCD [10]. Moreover, a single family has been described with pathogenic variants in the oral-facial-digital type 1 syndrome gene (*OFD1*) that are characterized by *X*-linked recessive mental retardation, macrocephaly, and PCD [12].

Transient electron microscopy (TEM) was once considered the gold standard for recognizing patients with PCD. However, approximately 30% of PCD cases have normal ciliary beating patterns, such as *DNAH11* and nexin-associated defects (e.g., CCDC65 and CCDC164), and thus would not be recognizable by TEM [3]. However, *DNAH11* has a distinct phenotype characterized by a hyperkinetic beating pattern and decreased beating amplitude. Patients have recurrent respiratory infections due to impaired ciliary motility and bronchiectasis. Many genes have been shown to be related to the inversion of symmetry. The variant in *DNAH11* is a likely explanation for situs inversus and recurrent infections in our patient. Pathogenic variants in the *DNAH11* gene do not cause obvious ultrastructural defects identifiable by TEM. Therefore, *DNAH11* could be overlooked if the diagnosis is established solely on TEM and further tests. Particularly, genetic testing is required to identify such pathogenic variants [3, 13]. In our patient, genetic testing confirmed the final diagnosis, too. However, it should be considered that in approximately 35% of PCD patients, the genes are not discovered. Therefore, precise clinical evaluation and other differential diagnoses are of great importance [14]. Until now, 39 genes have been identified as causing PCD, and any of the >200 genes involved in ciliary function could lead to PCD [15], but so far, only 65% of PCD patients have been diagnosed with genetic testing. In conclusion, genetic testing should be considered as part of the diagnostic process as the most reliable test in patients with PCD [14]. Gene sequencing for PCD is currently limited to the most prevalent alleles, including *DNAH5, DNAI1*, and *DNAH11*, in genetic panels; however, some companies provide whole exome or selective sequencing for PCD [5].

There are also some other criteria for PCD diagnosis. Levels of nasal nitric oxide (nNO) are low in PCD patients (<77 nl/min, normal range: 125 to 867 nl/min; mean, 287 nl/min); therefore, it can be a helpful test for PCD [16]. Patients with CF also show low levels of nNO, so it must be considered in the differential diagnosis of PCD and should be ruled out in workups [17]. Biopsies of respiratory epithelium could be used for ciliary motility evaluation to confirm PCD diagnosis; however, this approach is not sensitive enough to detect wide ranges of ciliary phenotype in this disease since the accuracy of high-speed video microscopy (HSVM) is limited and shows an overlap between PCD and normal patients in ciliary beat frequency [17]. Taken together, no single gold standard test exists to diagnose PCD. To make an early and definite diagnosis, a combination of technical investigations, including nasal nitric oxide (nNO), high-speed video microscopy analysis (HVMA), and TEM, is required.

In both Europe and North America, children with PCD have been diagnosed recently with a median age of 5.5 and 5.0 years, respectively [18]. Taking a focused history and making an early diagnosis are beneficial for the early diagnosis of PCD. PCD is among the top differential diagnoses and must be ruled out in the following conditions: (1) appropriate tests should exclude PCD in children with situs inversus totalis or any heterotaxic syndrome; (2) children with cerebral ventriculomegaly should have a PCD diagnosis excluded; (3) PCD should be excluded in babies with unexplained respiratory distress, especially when it is associated with other characteristics of PCD (respiratory manifestations are present in 65–87% of neonates, from mild transient tachypnoea to significant respiratory failure), if unexplained respiratory distress in term neonates is accompanied by atelectasis, the suspicion of PCD is raised; (4) children with chronic productive cough, unknown bronchiectasis, and severe upper airway disease should be referred for diagnostic testing for PCD; (5) males with immotile sperm should be considered for diagnostic testing for PCD; and (6) females with recurrent ectopic pregnancy should be considered for referral for diagnostic testing, especially if there are other associated symptoms of PCD [10]. The association of infertility with PCD is because of the similarity between the structure of the sperm flagella and the ultrastructure of cilia. Infertility in women with PCD is due to dysfunction of the cilia in the fallopian tubes [7]. Some medical disorders and phenotypes that may coexist with PCD include complex congenital heart disease, laterality defects, retinitis pigmentosa, hydrocephalus, pectus excavatum, and scoliosis [16]. Approximately 50% of PCD patients present situs anomalies that could be associated with congenital cardiac diseases, and in our patient, situs inversus was a clue [7]. Schultz et al. reported two Finnish families with clinical picture of PCD, including chronic wet cough, otitis media, rhinosinusitis, and situs inversus. Diagnostic tests were done, and two likely pathogenic variants of the *DNAH11* gene were identified (c.2341G > A, p. (Glu781Lys) and c.7645 + 5G > A) [4]. Another study conducted by Xia et al. reported a Chinese family with heterotaxy and congenital heart disease (CHD). Exome sequencing and sanger sequencing in a nonconsanguineous Han Chinese family with heterotaxy and CHD and 200 unrelated healthy subjects (control group) identified compound heterozygous variants (c.3426_1G > A and c.4306_C > T) in the *DNAH11* gene, resulting in CHD and heterotaxy [19].

To date, no clinical treatments have been proven to improve ciliary dysfunction in PCD. Regular clearance techniques should be performed for patients with PCD to prevent pulmonary infection [20]. Routine management of PCD pulmonary manifestations includes twice-yearly clinic visits, assessment of lung microbiology and pulmonary function like airway clearance, preventive measures such as pneumococcal and influenza vaccines, instructions for infection control, and antibiotic use to control infection [16, 21]. Moreover, exposure to inflammatory triggers such as tobacco smoke should be prevented. Physical activity is recommended in all cases to improve the strength of the respiratory muscles [21].

In this study, we reported a novel homozygous nonsense variant in *DNAH11* in a 3-year-old boy with primary ciliary dyskinesia. This variation is a likely explanation for situs inversus and recurrent infection in the patient, who also shows recurrent respiratory tract infection due to impaired ciliary motility and bronchiectasis. This study expanded the spectrum of PCD syndrome pathogenic variants in Iran and highlighted the prominent role of genetic testing in the diagnosis of PCD-associated pathogenic variants, especially *DNAH11*. More studies should be performed to determine the most common causes of PCD syndrome in this country.

## Figures and Tables

**Table 1:** Summary of patient's lab results.

	Results	Normal range
CBC test		
WBC	11500/mm^3^	3500–11000
Neutrophil percentage	66%	40–60%
Lymphocyte percentage	33%	20–40%
Monocyte percentage	1%	2–8%
RBC	4.2 × 10^6^/mcl	4.2–6 × 10^6^/mcl
Hgb	10.6 g/dL	14–18 g/dL
Hct	31.9%	36–46%
MCV	75.95 fl	80–98 fl
MCH	25.24 pg	26–32 pg
MCHC	33.23 g/dL	32–36 g/dL
PLT	319 × 10^3^/mm^3^	150–450 × 10^3^/mm^3^
Inflammatory markers		
ESR	50 mm/hr	0–10 mm/hr
CRP	30 mg/L	0–10 mg/L
Sweat test		
Sodium	38 meq/L	<70 meq/L
Chloride	35 meq/L	<40 meq/L
Immunology test		
IgE	1115 IU/mL	<68 IU/mL

**Table 2:** Clinical presentation of PCD [10].

Antenatal	Situs inversus totalis or heterotaxy on antenatal ultrasound scanning, mild fetal cerebral ventriculomegaly

Neonatal	Neonatal respiratory distress, continuous rhinorrhoea, heterotaxy, and hydrocephalus

Childhood	Chronic productive or wet-sounding cough, recurrent atelectasis, pneumonia, atypical asthma that is nonresponsive to treatment, daily rhinitis, nasal polyps, chronic sinusitis in older children, otitis media, and hearing loss

Adolescence and adult life	Bronchiectasis, chronic mucopurulent sputum production is common, digital clubbing, pulmonary function tests usually show a progressive obstructive or mixed pattern, nasal polyposis and halitosis infertility in males (50%) due to immotility of spermatozoa, ectopic pregnancy, and subfertility in females

## Data Availability

No datasets were generated or analyzed during the current study.
